# A Case of Misdiagnosed Hepatic Sarcoidosis: Evaluating Ultrasound Resolution Microscopy for Differentiating Hepatic Sarcoidosis from Hepatocellular Carcinoma

**DOI:** 10.3390/diagnostics16020238

**Published:** 2026-01-12

**Authors:** Jie Zhang, Kazushi Numata, Jintian Zhang, Wenbin Zhang, Feiqian Wang

**Affiliations:** 1 Department of Ultrasound, The First Affiliated Hospital of Xi’an Jiaotong University, No. 277 West Yanta Road, Xi’an 710061, China; zhangjiehhmz219@163.com; 2 Numata Clinic, Bandobashi 4-56, Akebono-cho, Naka-ku, Yokohama 231-0057, Japan; kz-numa@urahp.yokohama-cu.ac.jp; 3 Department of Cardiovascular Surgery, The First Affiliated Hospital of Xi’an Jiaotong University, Xi’an 710061, China; 18182605068@stu.xjtu.edu.cn; 4 VINNO Technology Company Ltd., Suzhou 215100, China; zhangwenbin3@hotmail.com

**Keywords:** hepatic sarcoidosis, hepatocellular carcinoma, ultrasound, ultrasound resolution microscopy, differential diagnosis

## Abstract

**Background and Clinical Significance:** Hepatic sarcoidosis is a benign lesion of unknown etiology. The gold standard for diagnosing hepatic sarcoidosis is histopathological examination. The symptoms and imaging findings of patients with hepatic sarcoidosis are often atypical, leading to misdiagnosis as hepatocellular carcinoma (HCC). Ultrasound resolution microscopy (URM) can overcome the diffraction limit, enabling fine visualization and quantitative analysis of the microvascular networks. This study aimed to provide new evidence for the differential diagnosis of these two diseases by comparing the URM parameters of hepatic sarcoidosis initially misdiagnosed as HCC with those of HCC. **Case Presentation:** A 67-year-old woman was admitted to the hospital due to upper abdominal pain for two weeks. Ultrasonography revealed a liver mass. The lesion was located in segment IV of the left hepatic lobe, was approximately 18 × 10 mm in size, and appeared hypoechoic. Contrast-enhanced ultrasound and enhanced magnetic resonance imaging both showed a “fast-in, fast-out” pattern, strongly suggesting HCC. The tumor markers were within the normal range. The patient underwent a laparoscopic left hepatic lobectomy. The histopathological diagnosis of the resected specimen was “hepatic sarcoidosis”. URM examination was performed during the preoperative diagnostic process. Subsequently, the URM parameters of the patient’s lesion were analyzed and compared with those of HCC. The results showed differences in multiple URM parameters, including microvascular flow velocity, diameter, microvascular density ratio, and vascular distribution, between this case of hepatic sarcoidosis and HCC. **Conclusions:** URM can quantitatively and multidimensionally evaluate the microvasculature of liver lesions, providing new reference data for the diagnosis and differential diagnosis of hepatic sarcoidosis.

## 1. Introduction

Sarcoidosis is a multisystem disease of unknown etiology that can develop in almost any organ, with incidence and prevalence varying by geographic region and ethnicity [1]. It affects both sexes nearly equally, though the peak incidence occurs later in women than in men [2]. Hepatic involvement represents one of the most common abdominal manifestations of sarcoidosis, occurring in 10–20% of patients. Though usually asymptomatic, it may occasionally present with nonspecific symptoms such as fatigue, malaise, weight loss, abdominal pain, or pruritus [3,4]. In its most severe form, hepatic sarcoidosis can progress to cirrhosis or portal hypertension [5,6]. In advanced cases liver transplantation becomes the definitive treatment, underscoring the importance of early diagnosis for prognosis and management. Although histopathology remains the gold standard for diagnosing hepatic sarcoidosis [7], needle biopsy carries inherent risks such as bleeding and infection. Imaging has long played a valuable role in the diagnosis and differential diagnosis of abdominal diseases. Ultrasound (US) serves as a first-line imaging modality for abdominal diseases. In a case report on hepatic sarcoidosis, Farina R et al. suggested that contrast-enhanced computed tomography (CT) is the most suitable technique for diagnosing hepatic sarcoidosis, as hepatic granulomas exhibit marked enhancement exclusively during the portal venous phase [8]. However, the imaging findings described in that report lack specificity, and current modalities remain insufficient for definitive diagnosis. Thus, new diagnostic approaches are urgently needed to identify hepatic sarcoidosis and to help distinguish it from hepatocellular carcinoma (HCC).

Microvascular architecture and hemodynamics serve as important indicators for diagnosing and evaluating many diseases [9]. Hepatic sarcoidosis is fundamentally an inflammatory condition characterized histologically by granulomas, which consist mainly of aggregated epithelioid histiocytes and multinucleated giant cells. In contrast, HCC is an aggressive malignant tumor. The two exhibit distinct patterns of angiogenesis. Angiogenesis, the formation of new vessels from pre-existing ones, is critical for malignant tumor growth [10]. Compared with normal tissues and benign lesions, malignant tumors often display disorganized vascular patterns with irregular branching and peripheral vascular penetration [11]. Ultrasound resolution microscopy (URM) is an emerging US based technique. This technique utilizes contrast-enhanced ultrasound (CEUS) to introduce micron-sized lipid-encapsulated microbubble (MB) into the bloodstream. High-frame-rate imaging is employed to detect and track the trajectories of these MBs (which simulate red blood cells). Based on the positions of the MBs, super-resolution images are generated, enabling the measurement of parameters such as blood flow velocity and the detailed mapping of the spatial architecture of the microvascular network [12,13]. Since URM is based on CEUS, it inherits the advantages of CEUS, including safety, non-invasiveness, convenience, low cost, and high reproducibility [14]. Moreover, when performing CEUS examination, the URM mode can be started up to complete the URM procedure. The stored URM data can be analyzed later, without increasing the patient’s financial burden or examination time. Most importantly, URM enables detailed quantification of multiple vascular characteristics by precisely detecting subtle contrast agent displacements across dozens of images per second and evaluating them with multiple parameters. The highest reported resolution for vascular structures is currently 5–10 μm [15]. Furthermore, it has been documented in the literature that URM achieved a detection limit of 40 µm in width for the main duct with a peak velocity of 15 mm/s, and 25 µm in width for secondary ducts [16]. In the field of liver research, this technique has been preliminarily applied to assess hepatic microperfusion in patients with focal liver lesions [17] (including HCC, hepatic hemangioma, and focal nodular hyperplasia), in rabbit models of liver cancer [18], in rats following liver resection and regeneration [19], and in rat models of liver cirrhosis [20]. For the differential diagnosis of benign and malignant space-occupying lesions, it has been initially utilized in tissues and organs such as the breast [21], kidney [22], and pancreas [22], yielding quantitative URM parameters with diagnostic value. In summary, the advantages of URM and its previously reported successful applications provide a rationale for employing URM in the differential diagnosis between hepatic sarcoidosis and HCC in the present study.

This study aims to characterize the microvascular flow patterns and URM parameter ranges in hepatic sarcoidosis, thereby offering a new reference for its differentiation from HCC.

## 2. Case Report

A 67-year-old woman was admitted with a two-week history of upper abdominal pain and the discovery of hepatic space-occupying lesions, and she underwent URM during preoperative diagnosis. The histopathological diagnosis from the surgical resection confirmed “hepatic sarcoidosis”. We retrospectively collected this patient’s data and obtained consent for the publication of relevant information. The report contains no personally identifiable details. All data collection, diagnosis, and treatment procedures adhered to the principles of the Declaration of Helsinki, under ethical approval from the Medical Ethics Committee of the First Affiliated Hospital of Xi’an Jiao Tong University on 6 June, 2023 (approval number XJTU1AF2023LSK-363).

**Figure 1:**
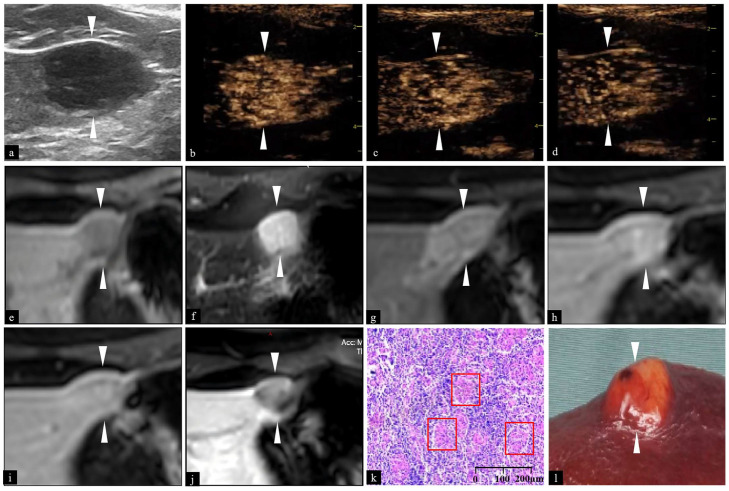
Imaging findings and pathological biopsy of the case. (**a**) Image obtained using the U5-15L linear array probe (frequency 5–15 MHz). (**b**–**d**) CEUS images demonstrating the lesion with homogeneous hyperenhancement in the arterial phase (**b**), followed by reduced enhancement in the portal phase (**c**), and further decreased enhancement in the delayed phase (**d**). (**e**,**f**) MRI revealing the lesion as hypointensity on T1-weighted imaging (**e**) and hyperintensity on T2-weighted imaging (**f**), respectively. (**g**–**j**) Enhanced MRI showing the lesion with homogeneous hyperenhancement, greater than the surrounding normal liver parenchyma in the arterial phase (**g**). Its enhancement decreases and becomes lower than the surrounding parenchyma in the portal phase (**h**) and delayed phase (**i**). The lesion exhibits markedly lower enhancement compared to the surrounding parenchyma in the hepatobiliary phase (**j**). (**k**) Hematoxylin and eosin (HE) staining of the tissue section. (**l**) Gross specimen of the liver and the lesion. White arrows in (**a**–**j**) indicate the lesion. The scale bar is in the lower right corner of (**k**). The red boxes in (**k**) indicates the granuloma. The white arrow in (**l**) indicates the lesion. CEUS, contrast-enhanced ultrasound; MRI, enhanced magnetic resonance; HE, hematoxylin and eosin.

Physical examination of the patient revealed no remarkable abnormalities. Laboratory tests indicated that routine tests of blood, urine, and stool, tumor markers, and infectious disease testing (hepatitis C, syphilis, and AIDS) were all within normal ranges. The positive hepatitis B surface antibody merely indicates a recovered state from past infection or an immune status following vaccination, not an active infection. The patient did not undergo an assessment for liver fibrosis, so the degree of liver fibrosis is unknown. Abnormal indicators of liver function, renal function, and electrolytes are presented in Table 1. As shown in Figure 1, the patient underwent grayscale US, CEUS, and contrast-enhanced magnetic resonance imaging (MRI) examinations in our hospital, all of which were diagnosed with HCC. The contrast agent used for the enhanced MRI was gadoxetic acid disodium (Gd-EOB-DTPA). Contrast-enhanced CT of the abdomen of the outpatient hospital showed that the lesion in the upper left outer lobe of the liver had a maximum diameter of about 21 mm, which was considered HCC. In the grayscale US images obtained at our institution, a hypoechoic lesion measuring approximately 18 × 10 mm was observed in segment IV of the left hepatic lobe. The lesion exhibited well-defined margins, an oval shape, and protruded beyond the hepatic capsule. Its internal echogenicity was heterogeneous. On CEUS, the lesion exhibited rapid contrast agent fast wash-in during the arterial phase, with enhancement occurring earlier than in the peri-lesional liver parenchyma, presenting as hyperenhancement. During the portal venous and delayed phases, the contrast agent washed-out rapidly. Meanwhile, its resolution occurred earlier than in the adjacent liver parenchyma, showing as hypoenhancement. On abdominal enhanced MRI, the lesion appeared as slightly hypointense on T1-weighted imaging and slightly hyperintense on T2-weighted imaging. Dynamic contrast-enhanced MRI revealed moderate to relatively high enhancement during the arterial phase. During the portal venous and delayed phases, a reduction in enhancement degree was shown, which was lower than that of the adjacent liver parenchyma. On the hepatobiliary phase, the lesion showed slight hypointensity. Both CEUS and enhanced MRI demonstrated a pattern of “fast wash-in and fast wash-out”, which is consistent with the imaging characteristics of HCC.

**Figure 2:**
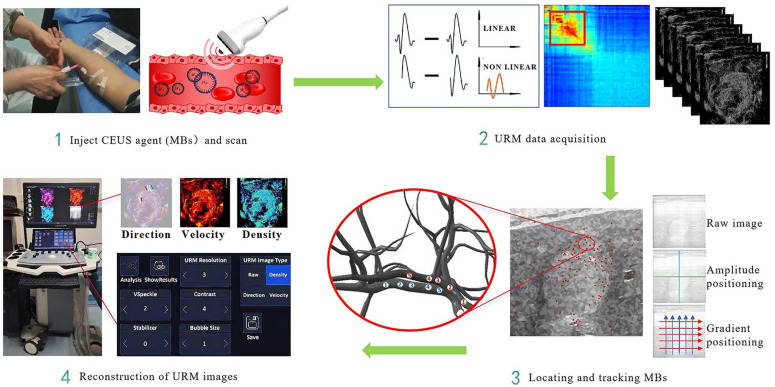
The URM operation process. A contrast agent, typically at low concentration, is first injected. Ultrafast CEUS then acquires raw MBs data at high frame rates. This method employs phase superposition to filter out tissue signals exhibiting linear oscillation, thereby isolating the MB signal. The red box highlights the filtered spectral bandwidth containing useful information. MBs are subsequently localized using amplitude, gradient, and motion compensation, and their trajectories are tracked. Finally, URM imaging maps, which depict microcirculation vessel structures, are generated from the motion trajectory, velocity, and orientation of MBs via super-algorithms. MB, microbubble; URM, ultrasound resolution microscopy; CEUS, contrast-enhanced ultrasound. This figure is adapted from Reference [13], with permission from the journal and the original authors.

In this case, CEUS was performed, followed by URM examination. The URM procedure was conducted using an ULTIMUS 9E US system (VINNO, Suzhou, China) equipped with a U5–15L linear array transducer (frequency range: 5–15 MHz). The contrast agent used was sulfur hexafluoride MBs for injection (SonoVue, Bracco, Shanghai, China), administered as a 1 mL bolus via the median cubital vein, followed by a 5 mL flush of 0.9% sodium chloride solution. The URM procedure is detailed in Figure 2. After initiating CEUS mode, the operator tapped the “Start” button on the US screen while injecting the contrast agent. Once contrast ingress appeared, the patient was instructed to hold their breath for 10 s before selecting the “URM Acquisition” button, as described in Reference [13]. The position, movement trajectory, and morphology of these microvessels were then located, tracked, and reconstructed to generate the original URM image. Subsequently, multiple parameters were measured from the static URM image, including the average microvessel diameter (μm), average and maximum blood flow velocity (mm/s), perfusion index, and density ratio (%). Density ratio (%) = Regions with microvessels determined based on microvessel density/Total area. This parameter reflects the abundance of blood supply within the region of interest and may be considered for differential diagnosis of disease and assessment of disease progression. The perfusion index was calculated as the Average Vascular Flow Velocity within the ROI multiplied by the Microvascular Density Ratio within the ROI. While velocity reflects blood flow speed and density indicates red blood cell quantity, their product more accurately represents the perfusion status. A higher perfusion index indicates regions with abundant and rapid blood flow. The dynamic bubble motion trajectory of hepatic sarcoidosis was saved as an AVI-format video, available in Supplementary Material S1. The Dynamic microbubble trajectorie of HCC is also provided in Supplementary Material S1 as a comparison.

URM values data for HCC were observed between hepatic sarcoidosis and HCC sourced from the literature. We only manually searched the PubMed database using the following strategy: (((super resolution ultrasound imaging [Title/Abstract]) OR (ultrasound resolution microscopy [Title/Abstract])) AND (hepatocellular carcinoma [Text Word]) on 24 October 2025. This search yielded three relevant articles published in the past decade [13,17,23]. One of these research applied a different ultrasound system than this study [17], while the other used murine models [23]. Therefore, this study only included one comparable reference [13].

The patient ultimately underwent a “laparoscopic left lateral hepatic lobectomy” without pre-resection biopsy. Based on guidelines [24], literature [25], hospital protocols, and patient preference, the hepatobiliary surgeon ultimately performed a segmental hepatectomy without prior biopsy.

Afterwards, the specimen was sent for pathological examination. Intraoperative rapid frozen section pathological examination was performed, followed by routine pathological examination of the resected tissue. Both pathological results indicated hepatic sarcoidosis. The gross specimen image and the histopathological photomicrograph are shown in Figure 1.

## 3. Discussion

Both CEUS and enhanced MRI in this case demonstrated the “fast wash-in and fast wash-out” pattern. This pattern is also a typical feature of HCC on both CEUS and enhanced MRI, characterized by marked enhancement in the arterial phase followed by decreased enhancement in the portal venous and delayed phases [26,27]. This similarity in enhancement patterns contributed to the misdiagnosis across various imaging modalities.

Given the combination of “no history of liver disease and all tumor markers negative”, multiple differential diagnoses were considered during the preoperative discussion. First, benign tumors included hepatocellular adenoma, focal nodular hyperplasia, and hepatic hemangioma. Patients with hepatocellular adenoma typically have no liver disease background and negative tumor markers, with enhanced scans often showing marked arterial phase enhancement that decreases in the portal venous and delayed phases. Nevertheless, hepatocellular adenoma usually occurs in women of childbearing age with a history of long-term oral contraceptive use, which was inconsistent with this patient. Histologically, almost all cases of focal nodular hyperplasia exhibit a central scar. The radiological characteristics of a central scar typically show low enhancement in the arterial and portal venous phases but high enhancement in the delayed phase, which did not match the imaging findings in this case. While most hepatic hemangiomas exhibit peripheral nodular enhancement in the arterial phase, the enhancement pattern of small hemangiomas is not consistent with this description. Their characteristic enhancement is uniform across the arterial, portal venous, and delayed phases. This pattern differs from the enhancement features observed in the present case. Second, malignant tumors considered included HCC and hepatic metastases. Despite negative tumor markers and no history of cirrhosis, HCC has a high occurrence rate, and its enhancement pattern on imaging was highly consistent with this case. No other primary lesions were detected on the patient’s chest and abdominal CT scans. Furthermore, hepatic metastases demonstrate rapid enhancement in the arterial phase, with the enhancement intensity beginning to decrease as early as the late arterial or portal venous phase, resulting in very low enhancement in the delayed phase, presenting as a “hollow sign”.

**Figure 3:**
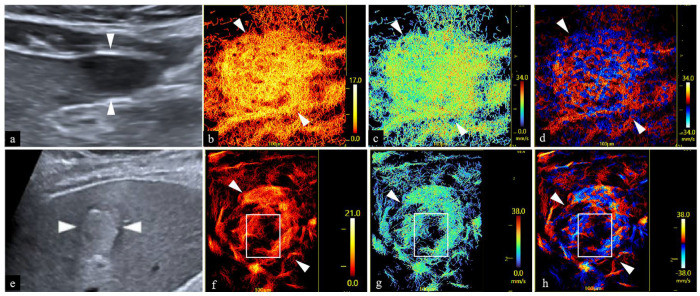
Microvascular images of this case and HCC using URM imaging. (**a**) Grayscale image of the present case obtained using the S1–8C convex array probe (center frequency: 3.2 MHz). (**b**–**d**) The lesion in this case demonstrated fine and homogeneous vasculature on the density map (**b**), velocity map (**c**), and direction map (**d**), with uniform perfusion of the contrast agent. (**e**) Grayscale image of HCC obtained using the S1–8C convex array probe (center frequency: 3.2 MHz). (**f**–**h**) The HCC lesion exhibited heterogeneous vasculature with thick branching vessels on the density map (**f**), velocity map (**g**), and direction map (**h**), accompanied by non-uniform contrast agent perfusion and the presence of perfusion defects. White arrows in (**a**–**h**) indicate the lesion. The white boxes in (**f**–**h**) denote the area of contrast agent perfusion defect. Figures (**e**–**h**) are reproduced from Reference [13] with permission from the journal and the original authors. HCC, hepatocellular carcinoma; URM, ultrasound resolution microscopy.

The URM density map, velocity map, direction map, and the URM image of HCC obtained in this case are shown in Figure 3. The HCC image was reproduced from reference [13], with permission from the journal and the original authors. A comparison of parameter values is provided in Table 2. The following observations were made. First, in the lesion area of hepatic sarcoidosis, the vascular diameters were small and uniform, with homogeneous perfusion of contrast agent and no definite perfusion defects. In contrast, the HCC lesion area exhibited non-uniform vascular diameters. In addition, the perfusion of contrast agent was heterogeneous. In details, the HCC lesion appeared as coarse branching vessels, patchy areas of clustered perfusion, and small regions with no contrast agent perfusion. A detailed comparison is presented in Table 3. Second, significant differences in URM values were observed between hepatic sarcoidosis and HCC. The diameter and density ratio of microvessels were lower in hepatic sarcoidosis than in HCC, whereas the microvascular blood flow velocity and vascular perfusion index were higher in hepatic sarcoidosis than in HCC.

We analyzed the mechanisms of blood supply formation in hepatic sarcoidosis and HCC, revealing that the two diseases share similar imaging manifestations but arise from different underlying causes. HCC is widely recognized as a highly vascularized heterogeneous tumor. Its growth, invasion, and metastasis depend on angiogenesis within the tumor. Normal liver tissue has a dual blood supply. The portal vein accounts for 75–80%, while the hepatic artery supplies only 20–25%. The “fast wash-in and fast wash-out” pattern observed in HCC on enhanced MRI and CEUS primarily stems from its unique angiogenic mechanisms and the physiological basis of dual hepatic blood supply [30]. In the liver architecture, the hepatic artery, portal vein, and bile duct run in parallel, collectively termed the portal triad. HCC tumor cells disrupt the portal triads, leading to reduced vascular supply and resultant intralesional hypoxia. Hypoxia stimulates angiogenic signaling, promoting the formation of new arterial branches within the lesion. Unlike the original distribution, these newly formed arteries are not accompanied by portal veins or bile ducts and are thus referred to as unpaired arteries [31]. As HCC progresses, the portal triads within and around the lesion nearly disappear. As compensation, the number of unpaired arteries increases significantly [32,33]. Consequently, HCC lesions rely almost exclusively on unpaired arteries for blood supply, whereas normal liver parenchyma is predominantly perfused by the portal vein [34,35]. Therefore, HCC lesions show pronounced enhancement in the arterial phase, while the normal parenchyma, supplied mainly by the portal vein, exhibits only mild enhancement. In the portal venous phase, a large amount of contrast agent enters via the portal vein, leading to marked enhancement of the liver parenchyma. Meanwhile, HCC shows lower enhancement than the parenchyma due to wash-out of the arterial-phase contrast agent and the lack of portal venous drainage [31]. In summary, HCC exhibits the specific “fast wash-in and fast wash-out” sign with rapid contrast clearance.

In contrast, CEUS findings of hepatic sarcoidosis typically show nodular enhancement in the late arterial and late portal phases with progressive hypo-enhancement [36]. In this case, however, CEUS demonstrated features resembling HCC. We attempted to analyze the possible reasons. Sarcoidosis is a complex immune-mediated disease triggered by genetic susceptibility, abnormal adaptive immunity in response to various antigens, and environmental factors [37]. Its development culminates in the formation of granulomas in affected organs or tissues. Granulomatous disease is not merely an inflammatory condition but a specific and classic manifestation of chronic inflammation. According to the literature, chronic inflammation induces extensive vascular changes, including increased vascular permeability, endothelial cell activation, and angiogenesis [38]. Neovascularization increases intralesional blood flow, facilitating substantial contrast inflow and resulting in marked enhancement in the arterial phase. As the contrast agent washes out, the lesion gradually becomes hypo-enhanced.

This case indicates that CEUS, contrast-enhanced CT, and enhanced MRI cannot reliably differentiate hepatic sarcoidosis from HCC. Nevertheless, microvascular flow imaging by URM revealed differences between the two diseases. We analyzed the significance of URM-derived microvascular maps and multiparameter values in evaluating hepatic sarcoidosis from a histological perspective, summarized in the following four points. First, although both HCC and chronic inflammation involve angiogenesis, the characteristics of the newly formed vessels differ. In chronic inflammation, angiogenesis is often transient and localized to inflammatory regions, meeting the demands of inflammatory cell infiltration and tissue healing. These vessels are structurally closer to normal physiological vessels and rarely exhibit thick branching. In the early stages of granuloma formation, inflammatory stimuli promote proliferation and migration of vascular endothelial cells, leading to new vessel formation. However, as the granuloma matures and stabilizes, the rate of angiogenesis gradually slows and eventually plateaus. In contrast, angiogenesis in HCC is a continuous and expanding process. As the tumor grows, vascular endothelial cells become irregularly arranged with incomplete pericyte coverage [29]. The vascular network becomes dilated, tortuous, and disorganized. These factors contribute to a higher microvascular density and diameter in HCC compared to hepatic sarcoidosis [2]. Second, the vascular network in HCC is chaotic and lacks the organized hierarchy of arterioles, capillaries, and venules, resulting in poor tissue perfusion. Immature vessels with inadequate pericyte coverage further increase the risk of hypoperfusion [28]. This leads to heterogeneous contrast perfusion in HCC. In sarcoidosis, the vascular architecture more closely resembles physiological vessels, with regularly arranged endothelial cells and intact pericyte coverage, making heterogeneous perfusion less likely. Third, due to the abundant and sustained release of pro-angiogenic factors in the tumor microenvironment, newly formed vascular networks in HCC often fail to mature and undergo effective pruning. The resulting heterogeneity in vascular caliber contributes to the presence of thick, branching vessels in HCC [39]. In contrast, sarcoidosis is characterized mainly by fine, tortuous, capillary-like structures. Fourth, in HCC, fibrosis, vascular compression, or thrombosis in some tumor regions can increase vascular resistance and affect blood flow velocity [40,41]. Such phenomena are uncommon in sarcoidosis. The interpretable URM parameter features suggest that URM may serve as an auxiliary diagnostic tool for hepatic sarcoidosis in the future.

This study has several limitations. First, although routine tests such as liver and kidney function and electrolytes showed mild abnormalities, they were not diagnostically informative (Table 1). Studies indicate that sarcoidosis is often associated with elevated serum angiotensin-converting enzyme levels [1]. Due to the low occurrence rate of hepatic sarcoidosis, this condition was not considered in the preoperative differential diagnosis. Consequently, specific laboratory tests relevant to sarcoidosis were not performed prior to surgery. In the future, preoperative evaluation of challenging or atypical hepatic space-occupying lesions should be as comprehensive as possible. Second, there is limited research on angiogenesis features in chronic inflammation, particularly in granulomatous diseases, restricting the data available for comparison. Third, as URM is a novel technique and has not yet been adopted as a routine diagnostic procedure in our hospital, it is not used as a diagnostic criterion by clinicians. Consequently, it is difficult to collect large-scale data or perform comparative analyses based on this method. And the URM index is protocol- and equipment-dependent; therefore, more cases need to be accumulated under standardized protocols before clinical application can be considered. Finally, due to misdiagnosis, the patient in this case underwent left lobe resection, resulting in the loss of follow-up data for the hepatic sarcoidosis lesion. Therefore, we could not observe changes in URM parameters during the progression of sarcoidosis, which would have provided valuable diagnostic insights. Nevertheless, given the rarity of hepatic sarcoidosis and the novelty of URM, the diagnostic analysis using URM in this case remains particularly valuable.

The URM technology employed in this study enables quantitative analysis, thereby compensating for the limitations of qualitative CEUS examinations. It demonstrates distinct differential characteristics in the differential diagnosis of HCC. This technique is considered to provide valuable reference for subsequent research on hepatic sarcoidosis.

## 4. Conclusions

Based on CEUS, URM not only retains the advantages of CEUS—such as safety, non-invasiveness, convenience, and high reproducibility—but also provides a variety of precise quantitative microvascular parameters. This offers new reference value for the differential diagnosis of hepatic sarcoidosis and HCC, suggesting its potential as an auxiliary tool for diagnosing challenging liver cases in the future.

## Figures and Tables

**Table 1:** Laboratory Indicators of This Case.

Code Name	Name	Outcome	Reference Range	Unit
DBIL	Direct bilirubin	4.9	0–3.4	μmol/L
TP	Total protein	63.0	65–85	g/L
ALB	Albumin	37.1	40–55	g/L
Mg	Magnesium	1.12	0.75–1.02	mmol/L
CO_2_	Carbon Dioxide Binding Capacity	21.8	22–29	mmol/L

**Table 2:** URM Features of HCC in Reference [13] and Hepatic Sarcoidosis in Our Case ^1^.

Disease	Mean Diameter of the Microvessels (μm)	Microvascular Blood Flow Velocity (Average/Maximum) (mm/s)	Microvascular Perfusion Index	Proportion of Microvascular Density (%)
Hepatic sarcoidosis	144.33	15.98/34.00	12.65	75
HCC	300.00 ± 70.00	5.29/16.71	2.12	80

^1^ HCC, hepatocellular carcinoma; URM, ultrasound resolution microscopy.

**Table 3:** Comparison of Hepatic Sarcoidosis and HCC Blood Vessels ^1^ [28,29].

Disease	Pathological Essence	Vascularity	Diameter Difference	Vascular Manifestations of URM
Hepatic sarcoidosis	Granuloma	Uniform	Fine and uniform	Fine uniform capillary network
HCC	Malignancy	Uneven	There are coarse branches	Dendritic coarse blood vessels

^1^ HCC, hepatocellular carcinoma; URM, ultrasound resolution microscopy.

## Data Availability

The raw data supporting the conclusions of this article will be made available by the authors without any undue reservation.

## Supplementary Materials

The following supporting information can be downloaded at: https://www.mdpi.com/article/10.3390/diagnostics16020238/s1, S1: The Dynamic microbubble trajectorie of Hepatic Sarcoidosis and HCC.
